# Effect of foot immersion and neck cooling on renal, intestinal, immune and inflammatory markers in older adults exposed to extreme heat

**DOI:** 10.1113/EP093094

**Published:** 2025-11-03

**Authors:** Thomas McCarthy, Ben J. Lee, James J. McCormick, Kelli E. King, Emma R. McCourt, Luana C. Main, Robert D. Meade, Glen P. Kenny

**Affiliations:** ^1^ Occupational and Environmental Physiology Group, Centre for Physical Activity, Sport, Exercise Sciences Coventry University Coventry UK; ^2^ Human and Environmental Physiology Research Unit, School of Human Kinetics University of Ottawa Ottawa Canada; ^3^ Clinical Epidemiology Program Ottawa Hospital Research Institute Ottawa Ontario Canada; ^4^ Institute for Physical Activity and Nutrition (IPAN), School of Exercise and Nutrition Sciences Deakin University Geelong Australia

**Keywords:** acute kidney injury, ageing, cooling interventions, heat stress, immune activation, intestinal epithelial injury, systemic inflammation

## Abstract

Older adults with reduced thermoregulatory capabilities are increasingly at risk of heat‐related pathophysiological outcomes (e.g., acute kidney injury, heatstroke) due to increasingly frequent, prolonged and intense heatwaves. Foot immersion and neck cooling have been proposed as practical, non‐electrical cooling strategies for protecting older adults during heatwaves, though evidence supporting their efficacy is limited. This study evaluated the effect of foot immersion with or without neck cooling on systemic proteins associated with acute kidney injury (NGAL, KIM‐1), intestinal enterocyte damage (IFABP), immune activation (sCD14) and systemic inflammation (IL‐6, TNF‐α, CRP) in older adults. Seventeen participants (nine females; median [IQR] age 72 [69–74] years) completed three randomized 6‐h passive heat exposures (38°C, 35% relative humidity) with no‐cooling, foot immersion in 20°C water, or foot immersion with neck cooling via wet towels. Thermal and cardiovascular strain were measured throughout exposures, with venous blood samples collected pre‐ and post‐exposure. Body core temperature increased by ∼1.1°C (*P *< 0.001) with no changes in any measured systemic proteins (all *P *> 0.05) across conditions. Foot immersion with or without neck cooling modestly reduced heart rate, mean skin temperature, whole‐body sweat rate and fluid consumption (*P *< 0.05), but had no effect on body core temperature or systemic protein concentrations (all *P *> 0.05) relative to no‐cooling. These findings do not support the efficacy of these interventions for mitigating hyperthermia in older adults during heatwaves. Further research is warranted to evaluate their efficacy for protecting against heat‐related acute kidney injury, intestinal enterocyte damage, immune activation, or systemic inflammation under more severe exposure conditions.

## Abstract (highlights)

- **What is the central question of this study?**
  Does foot immersion with or without neck cooling mitigate increases in systemic proteins associated with acute kidney injury (NGAL, KIM‐1), intestinal enterocyte damage (IFABP), immune activation (sCD14), or systemic inflammation (IL‐6, TNF‐α, CRP) in older adults during a 6 h simulated heatwave exposure (38°C, 35% relative humidity)?
- **What is the main finding and its importance?**
  Systemic protein concentrations were unchanged relative to baseline and were not affected by foot immersion with or without neck cooling, alongside a ∼1.1°C increase in body core temperature across conditions. This study does not support the efficacy of these interventions, despite modestly reducing cardiovascular strain, for protecting older adults under the conditions studied.

## 1 INTRODUCTION

Extreme heat poses a significant health risk, with pathophysiological outcomes often quantified using biomarkers of renal injury, intestinal damage, immune activation and systemic inflammation (Lee et al., 2025a; Lee et al., 2025b; Lim, 2018; Mckenna et al., 2025). Older adults are particularly vulnerable due to age‐related declines in thermoregulatory function and the high prevalence of chronic conditions that exacerbate heat sensitivity (Meade et al., 2020). Given increasing frequency and severity of heatwaves, there is a pressing need for accessible, sustainable and non‐electrical cooling strategies to mitigate these risks. Foot immersion (i.e., immersing the lower limbs in cold water) has been identified as a potentially efficacious non‐electrical cooling intervention (Jay et al., 2021), owing to preliminary research demonstrating its ability to reduce whole‐body sweat rate and thermal discomfort in younger adults during 3 h of exposure to hot, humid conditions (40°C, 50% relative humidity) (Morris et al., 2019). More recently, when combined with neck cooling (i.e., applying cold wet towels to the neck), foot immersion was shown to have small–moderate effects on heart rate, sweat rate and mean skin temperature in older adults during 6 h of exposure to conditions simulating recent deadly heatwaves in North America (38°C, 35% relative humidity) (Meade et al., 2024).

While our previous work does not robustly support the efficacy of foot immersion and neck cooling, its focus on thermal and cardiovascular strain may have overlooked additional physiological responses associated with the pathogenesis of heat‐related maladies such as heatstroke and acute kidney injury (Ogden et al., 2020; Schlader et al., 2022). Older adults have been shown to experience increases in biomarkers associated with intestinal enterocyte damage (e.g., intestinal fatty acid binding protein [IFABP]; Foster et al., 2023; Lee, Flood, Galan‐Lopez, et al., 2024; Lee, Russell, et al., 2024; Lee et al., 2025b, 2025a) and renal tubular stress/damage (e.g., neutrophil gelatinase associated lipocalin [NGAL], kidney injury molecule 1 [KIM‐1]; Lee, Flood, Russell, et al., 2024) following exposures to simulated heatwave conditions that induced thermal and cardiovascular strain, potentially indicating increased risk for heatstroke and acute kidney injury, respectively (Chapman, Johnson, Parker, et al., 2020; Lim, 2018; Ogden et al., 2020; Schlader et al., 2022). Although apparent enterocyte damage rarely translated into elevated systemic proteins associated with immune activation (e.g., soluble cluster of differentiation 14 [sCD14]) or systemic inflammation (e.g., interleukin 6 [IL‐6], tumour necrosis factor alpha [TNF‐α], C reactive protein [CRP]) (Foster et al., 2023; Lee, Flood, Galan‐Lopez, et al., 2024; Lee, Russell, et al., 2024; Lee et al., 2025b, 2025a) – responses linked to the pathogenesis of heatstroke (Zhang et al., 2024) – it remains unclear whether the small–moderate effects of foot immersion and neck cooling observed in our previous work (Meade et al., 2024) could meaningfully mitigate intestinal or renal stress/damage during heat stress, thereby potentially reducing older adults’ risk for heatstroke or acute kidney injury, respectively (Chapman, Johnson, Parker, et al., 2020; Lim, 2018; Ogden et al., 2020; Schlader et al., 2022).

To date, however, no study has evaluated whether foot immersion and neck cooling protect against heat‐related acute kidney injury, intestinal enterocyte damage, immune activation or systemic inflammation in older adults. Addressing this knowledge gap is necessary to evaluate the efficacy of these cooling interventions for use among older adults, particularly those without access to electrical‐cooling systems, during heatwaves. Therefore, this exploratory study evaluated the effect of foot immersion with and without neck cooling on circulating concentrations of NGAL, KIM‐1, IFABP, sCD14, IL‐6, TNF‐α and CRP in older adults following 6 h of exposure to conditions simulating recent North American heatwaves (38°C, 35% relative humidity).

## 2 METHODS

This study was registered at ClinicalTrials.gov with identifier NCT05601713.

### 2.1 Ethical approval

Ethical approval for this single‐site, laboratory‐based, randomized crossover trial (NCT05601713), which adhered to the *Declaration of Helsinki*, was obtained from the University of Ottawa Health Sciences and Science Research Ethics Board (H‐11‐206234). All participants provided written and informed consent preceding their enrolment in this study.

### 2.2 Participants

**FIGURE 1:**
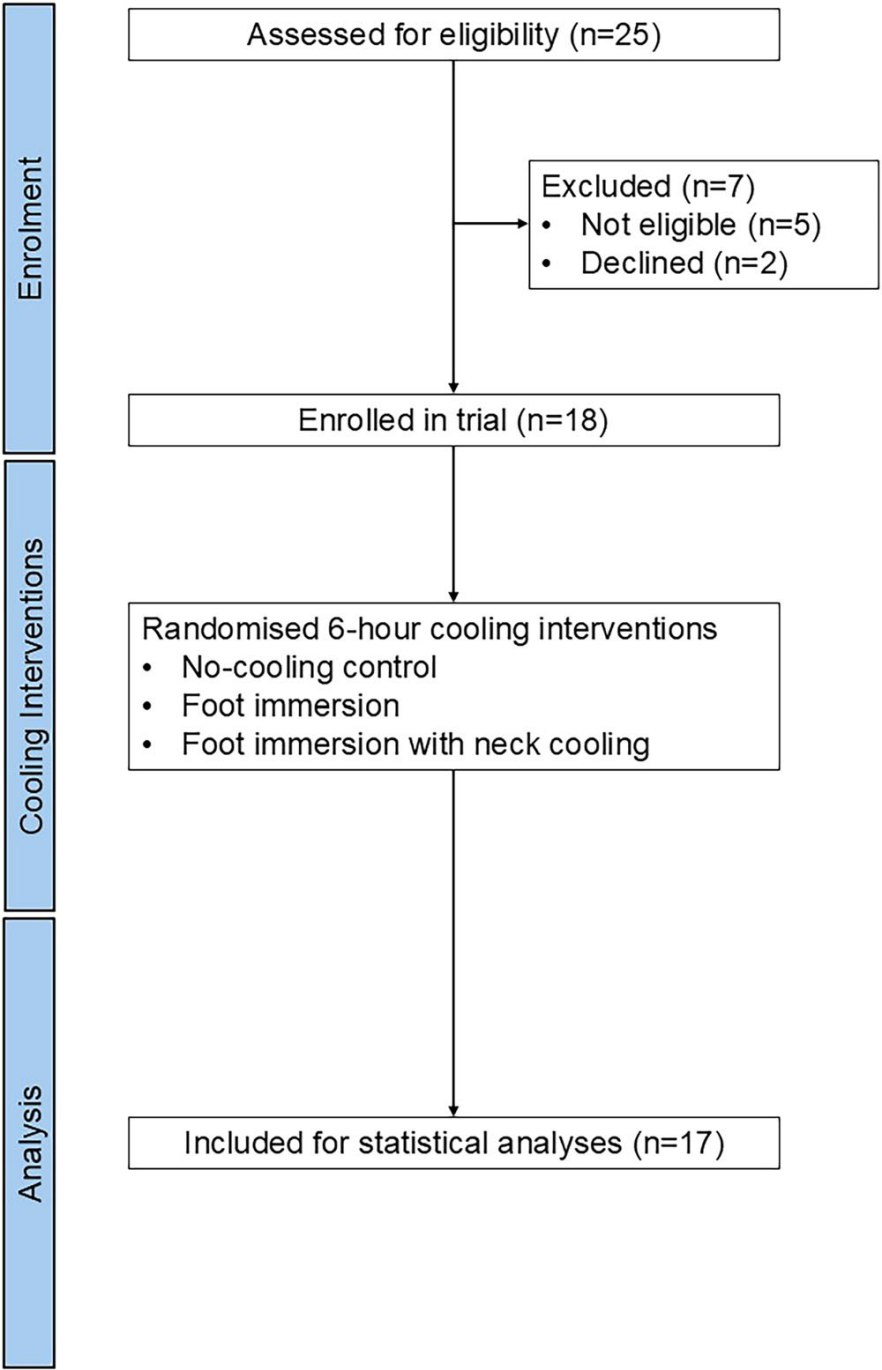
Flow of participants through the study. CONSORT diagram illustrating participant enrolment, randomization and analysis. Of the 25 individuals screened for eligibility, 18 older adults were enrolled. All but one participant completed three randomized 6 h heat exposures with no cooling (control, CON), foot immersion in 20°C water, or foot immersion with a 20°C wet towel around the neck.

Prospective participants were screened for eligibility based on inclusion and exclusion criteria. Male and female adults aged 65–85 years were eligible to participate if they were non‐smoking, English or French speaking, able to provide informed consent, and did not meet any of the following exclusion criteria: physical restriction (e.g., due to disease: intermittent claudication, renal impairment, active proliferative retinopathy, unstable cardiac or pulmonary disease, disabling stroke, severe arthritis, etc.); use of or changes in medication judged by the patient or investigators to make participation in this study unadvisable; peak aerobic capacity (${\dot{V}}_{O_{2}\mathrm{peak}}$) exceeding the 50th percentile of age‐ and sex‐specific normative values published by the American College of Sports Medicine; and cardiac abnormalities identified in screening. Those diagnosed with or taking medications for hypertension and/or type 2 diabetes who otherwise met the inclusion criteria were not excluded from participation. A CONSORT diagram depicting the flow of participants through this study is presented in Figure 1.

Seventeen participants (9 females; median [IQR] age: 72 [69–74] years; height 168 [159–172] cm; body mass: 71.2 [55.5–77.5] kg; body mass index: 24.8 [22.0–26.6] kg/m^2^; body surface area 1.8 [1.6–1.9] m^2^) completed all three of this study's experimental trials and were therefore included for statistical analyses. The physical characteristics of these participants are displayed in Table 1.

**TABLE 1:** Physical characteristics of participants included for statistical analyses.

Variable	All participants (*n* = 17)
Age (years)	72 (69–74)
Sex (*n* (%))	
Females	9 (53%)
Males	8 (47%)
Smoking status (*n* (%))^a^	
Never	11 (65%)
Past	6 (35%)
Habitual physical activity (min/week)^b^	180 (60–240)
Types of physical activity (*n* (%))^b^	
Walking	13 (76%)
Jogging, biking, swimming	7 (41%)
Aerobics, floor exercises	3 (18%)
Organized sports	3 (18%)
Peak aerobic capacity (${\dot{V}}_{O_{2}\mathrm{peak}}$) (mL/kg/min)^c^	25 (22–27)
ACSM ${\dot{V}}_{O_{2}\mathrm{peak}}$ percentile (%)^c^	17 (13–35)
Using prescribed medications (*n* (%))^d^	15 (88%)
Haemoglobin A_1c_ (%)	5.6 (5.4–5.8)

*Note*: Values are median and interquartile range (IQR) or no. participants (%). Reported medications included: antihypertensives (e.g., ACE inhibitors, angiotensin II receptor blockers, diuretics, calcium channel blockers; *n* = 4), antidepressants (e.g., serotonin and noradrenaline reuptake inhibitors, selective serotonin reuptake inhibitors, *n* = 3), metformin (*n* = 2), statins (*n* = 6), topical creams/ointments (*n* = 1), hormonal replacements (*n* = 2), corticosteroids (*n* = 2), bronchodilators (*n* = 2), antihistamines (*n* = 1) and medications treating gastrointestinal reflux (*n* = 2), hypoactive thyroid (*n* = 2), benign prostatic hyperplasia (*n* = 2), occasional pain (e.g., for headaches and joint pain; *n* = 2), osteoporosis (*n* = 2), gout (*n* = 1), hair loss (*n* = 1), antibiotics (*n* = 1), and sodium–glucose cotransporter inhibitors (e.g., blood sugar management; *n* = 1).

^a^ Smoking status determined via participant self‐report. Prospective participants were excluded if they were currently smoking. All past smokers quit ≥19 years prior to participation.

^b^ Participant self‐reported physical activity level determined via the Canadian Society for Exercise Physiology Get Active Questionnaire (GAQ). The types of physical activity performed were determined via the Kohl Physical Activity Questionnaire.

^c^ Prospective participants peak aerobic power (${\dot{V}}_{O_{2}\mathrm{peak}}$) was assessed during an incremental cycling test to volitional fatigue. Volunteers were excluded if their ${\dot{V}}_{O_{2}\mathrm{peak}}$ exceeded the 50th percentile of age‐ and sex‐specific normative data published by the American College of Sports Medicine (ACSM).

^d^ Participant prescription medication use determined via self‐report. Some participants reported medications that have been suggested to increase heat vulnerability (e.g., antidepressants) or are taken to treat health conditions known to reduce heat tolerance (e.g., type 2 diabetes, heart conditions).

### 2.3 Preliminary screening

Before the screening visit, prospective participants completed the Canadian Society for Exercise Physiology (CSEP) Get Active Questionnaire (GAQ; Tremblay et al., 2011) and the American Heart Association Pre‐Participation Screening Questionnaire, which were examined and used to determine their eligibility to participate. The GAQ and Kohl Physical Activity Questionnaire (Kohl et al., 1988) were also used to evaluate participants habitual physical activity levels and types of physical activity.

Upon arrival for the screening visit, which took place in temperate ambient conditions (∼22°C), participants were familiarized with all procedures and measurements involved in this study. Participants then provided written and informed consent to undergo exercise testing. In line with the American Heart Association's guidelines (Muntner et al., 2019), participants sat quietly for ≥15 min before their resting arterial blood pressure was measured in triplicate (∼1 min between measures) using automated oscillometry of the brachial artery (UM‐211, A&D Medical, Tokyo, Japan). Participants’ height and body mass were then measured using a physician stadiometer (model 2391, Detecto, Webb City, MO, USA) and a high‐performance weighing terminal (CBU150X, Mettler Toledo Inc., Mississauga, ON, Canada), respectively, for the calculation of body mass index and body surface area. Participants then completed an incremental exercise test to volitional fatigue on a semi‐recumbent cycle ergometer (Corival, Lode B.V., Groningen, Netherlands) while a certified exercise physiologist monitored heart rate and screened for cardiac abnormalities using 12‐lead echocardiogram. Throughout exercise testing, oxygen consumption was measured using a metabolic gas analysis system (MCD Medgraphics Ultima Series, MGC Diagnostics, MN, USA), and peak oxygen consumption (${\dot{V}}_{O_{2}\mathrm{peak}}$) was quantified using indirect calorimetry. Participants whose ${\dot{V}}_{O_{2}\mathrm{peak}}$ exceeded the 50th percentile of age‐ and sex‐specific normative values were excluded from further testing.

### 2.4 Experimental design

All except one of the 18 participants who were enrolled following the screening session completed three randomized experimental trials, each of which was separated by ≥4 days (median [IQR] duration between each session: 6 [6, 7] days) and took place at the University of Ottawa's Human and Environmental Physiology Research Unit (HEPRU; Ottawa, Ontario, Canada), between September 2022 and April 2023. The trials were conducted during non‐summer months to minimize the potential for natural heat acclimatization, which typically occurs during summer months, and because most cases of heat‐related morbidity and mortality occur more during early summer months when individuals are less likely to be heat acclimatized (Gasparrini et al., 2016).

Each experimental trial involved a 6 h exposure to conditions simulating recent deadly heatwaves in North America (38°C, 35% relative humidity; Henderson et al., 2022) with one of the following three experimental conditions applied in a randomized order: (1) no cooling intervention (control); (2) immersion of the foot to mid‐calf in ∼100 L of ∼20°C tap water during the last 40 min of each hour, with the tap water in the 100 L vessel replenished during the first 20 min of each hour (foot immersion); and (3) foot immersion with the addition of a hand towel (90 cm by 40 cm) soaked in ∼20°C tap water draped around the neck and re‐wet every 20 min (foot immersion and neck cooling).

Participants were instructed to abstain from strenuous physical activity and the consumption of alcohol for 24 h, and excessive caffeine consumption for 12 h preceding each laboratory visit. Prior to their arrival at the laboratory, participants were instructed to consume their normal breakfast and to bring a similar sized meal for their lunch during each experimental session. To facilitate euhydration upon arrival, participants were advised to consume ∼500 mL of water the night before and morning of each laboratory visit. Euhydration was verified upon arrival for each visit via the measurement of urine specific gravity (USG). If the threshold for hypohydration (USG: ≥1.025; Kenefick & Cheuvront, 2012) was exceeded, participants were provided with 400–500 mL of tap water, and USG was reassessed after ∼30 min. A questionnaire regarding heat exposure, physical activity and sleep habits, dietary intake, and general wellbeing was completed by participants during the baseline measurements period of each session (i.e., pre‐exposure). Participants were emailed this information ∼48 h before the subsequent session and were asked to replicate their previous activity patterns as closely as possible preceding that session. Light summer clothing (light shirt, shorts and sandals: ∼0.4 Clo) was worn by participants throughout experimental sessions.

### 2.5 Experimental protocol

Experimental sessions commenced between 07.00 and 09.00 h. Following the confirmation of euhydration (USG ≤1.025; Reichert TTS 400, Reichert, Depew, NY, USA), a measurement of nude body mass was recorded. Participants inserted a rectal thermocouple and dressed into light summer clothes (e.g., shorts and T‐shirt) before being transferred into a temperate room (22°C) to perform a specialized battery of cardiac autonomic response tests (see McCourt et al., 2024 or supplementary materials provided by Meade et al., 2024 for more detail). A ∼30 min period of seated rest followed, during which final instrumentation was completed, baseline measurements (rectal and skin temperatures, heart rate, arterial blood pressure, thermal sensation, and comfort) were recorded, and participants completed a series of questions regarding the previous days’ activity patterns (described in ‘Experimental design’). On completion, a baseline blood sample was collected from the antecubital vein.

Participants were then transferred to a climate‐controlled chamber to begin a 6 h exposure to 38°C, 35% relative humidity, with one of the three experimental conditions. Throughout these exposures, participants were permitted to drink tap water (∼15–20°C) ad libitum and were able to eat a self‐provided low water content meal (e.g., cheese sandwich) during the first 3 h of exposure.

Measurements of rectal temperature, skin temperature and heart rate were recorded continuously; and arterial blood pressure, thermal comfort, thermal sensation, fluid consumption, and body mass were recorded hourly throughout exposures. Following each measurement of body mass, participants were permitted to remain standing and/or perform light stretching for ≤5 min if desired. On completion of the 6 h exposure (including final measurements), a post‐exposure venous blood sample was taken before participants performed a second, identical battery of cardiac autonomic response tests. Thereafter, participants exited the chamber and recorded a final measurement of nude body mass before being de‐instrumented, provided with water and/or a sports drink, and dismissed from the laboratory, thus concluding the trial.

### 2.6 Outcome measurements and processing

#### 2.6.1 Body core temperature

For 14 of the 17 participants, rectal temperature was monitored continuously using a general‐purpose thermocouple temperature probe (Mon‐a‐therm General Purpose Temperature Probe, Mallinckrodt Medical Inc, St. Louis, MO) inserted ∼12 cm beyond the rectal sphincter. The remaining three participants who refused the rectal probe instead ingested temperature capsules (e‐Celcius, BodyCap, Caen, France) for the continuous monitoring of gastrointestinal temperature. Both rectal and gastrointestinal temperature data were used as an index of body core temperature and were analysed using averages from the last 15 min of each hour of exposure (i.e., minutes 45–60 of each hour).

#### 2.6.2 Skin temperature

Skin temperature data were collected using wireless temperature data loggers (DS1922L Thermochron, OnSolution Pty Ltd, Sydney, Australia) affixed to eight body sites (forehead, scapula, chest, upper arm, forearm, hand, thigh, calf) and sampling at 1 min intervals. Mean skin temperature was calculated using ISO 9886:2004 recommended weightings for the eight body sites (7% forehead, 17.5% right scapula, 17.5% upper left chest, 7% upper right arm, 7% right forearm, 5% left hand, 19% right anterior thigh, 20% left calf).

#### 2.6.3 Heart rate and blood pressure

Heart rate was measured in triplicate (>60 s between measures) at baseline and hourly at the end of each exposure using automated oscillometry of the brachial artery (UM‐211, A&D Medical, Tokyo, Japan), adhering to American Heart Association Guidance (Muntner et al., 2019).

#### 2.6.4 Fluid balance

Nude body mass was measured pre‐ and post‐exposure using a high‐performance weighing terminal (model CBU150X, Mettler Toledo Inc.). The change in nude body mass from pre‐ to post‐exposure, corrected for the mass of food and water consumed during the exposure, was used to estimate fluid loss via sweat and urination and is expressed as percentage change from baseline values (%). Whole‐body sweat rate was calculated as absolute fluid loss divided by exposure duration and is expressed as mL/h.

#### 2.6.5 Venous blood sampling and analysis

Venous blood samples (10 mL each) were drawn from the antecubital vein before and shortly after each 6 h heat exposure. To ensure plasma volume stability, participants rested in a slightly reclined seated position for ≥20 min before each blood sample was drawn. Upon collection, blood samples were transferred directly into Vacutainer tubes (5.4 mg K2EDTA, BD, Franklin Lakes, NJ) and serum separator tubes, respectively. Blood collected in Vacutainer tubes was mixed via inversion, and serum separator tubes were left standing for ∼15 min for blood to coagulate. Haemoglobin and haematocrit were measured in duplicate (Ac·T diff2, Beckman Coulter, Brea, CA, USA) using an aliquot of plasma; and plasma volume change (%) was calculated from average values using the method of Dill and Costill (1974). Following centrifugation of the remaining blood (1380 × relative centrifugal force, 10 min), plasma and serum were aliquoted and stored at −80°C for later analysis.

Enzyme‐linked immunosorbent assays (ELISA; R&D Systems, Minneapolis, MN) were used to analyse all serum and plasma samples in duplicate. In preparation for analysis, all samples were diluted at a ratio of 1:5 with phosphate‐buffered saline containing 1% bovine serum albumin, except for CRP, which was diluted 1:500. Assays for IFABP (DY3078), sCD14 (DY383), NGAL (DY1757), KIM‐1 (DY1750B), IL‐6 (DY206), TNF‐α (DY210), and CRP (DY1707) were conducted in line with the manufacturers’ standard sandwich ELISA protocol. Absorbance was measured using a BioTek SynergyMX plate reader (Agilent Technologies, Santa Clara, CA) at 450 nm, with a wavelength correction of 570 nm applied to account for potential non‐specific wavelength emissions. The respective inter‐ and intra‐plate coefficient of variation for each assay were recorded as follows: IFABP 3.02% and 3.72%; sCD14 4.29% and 5.72%; NGAL 5.50% and 7.16%; KIM‐1 3.12% and 3.52%; IL‐6 2.08% and 4.04%; TNF‐α 2.49% and 3.78%; and CRP 6.34% and 7.51%.

### 2.7 Statistical analysis

The sample size calculation for body core temperature, – the primary outcome of the larger project for which this study is part of – is presented within the original report from this project (Meade et al., 2024). As no formal power calculations were performed for secondary outcomes of the larger project (e.g., mean skin temperature, heart rate, whole‐body sweat rate, concentrations of IFABP, sCD14, NGAL, KIM‐1, TNF‐α, IL‐6 and CRP), this study's findings should be considered exploratory.

Statistical analyses were conducted in R (version 4.2.2, R Core Team). Main effects of condition, time and condition × time interactions were determined via mixed linear models, incorporating participant ID as a random intercept. The effect of cooling intervention (foot immersion, foot immersion with neck cooling compared with no‐cooling (control)) on all outcomes was analysed using linear mixed‐effects models, each including condition as a fixed effect, baseline values as a continuous covariate (to account for within‐ and between‐subject variability), and participant ID as a random intercept (to account for repeated measures due to the crossover design). Estimated marginal means were derived from baseline‐adjusted post‐exposure values, and pairwise comparisons were performed with the Bonferroni adjustment applied to control for the family‐wise error rate. Within‐condition data are reported as means ± SD; and between‐condition differences are reported as mean difference [95% confidence interval; lower, upper]. Statistical significance was set at *P *< 0.05.

## 3 RESULTS

### 3.1 Thermoregulatory, cardiovascular and fluid balance responses

**FIGURE 2:**
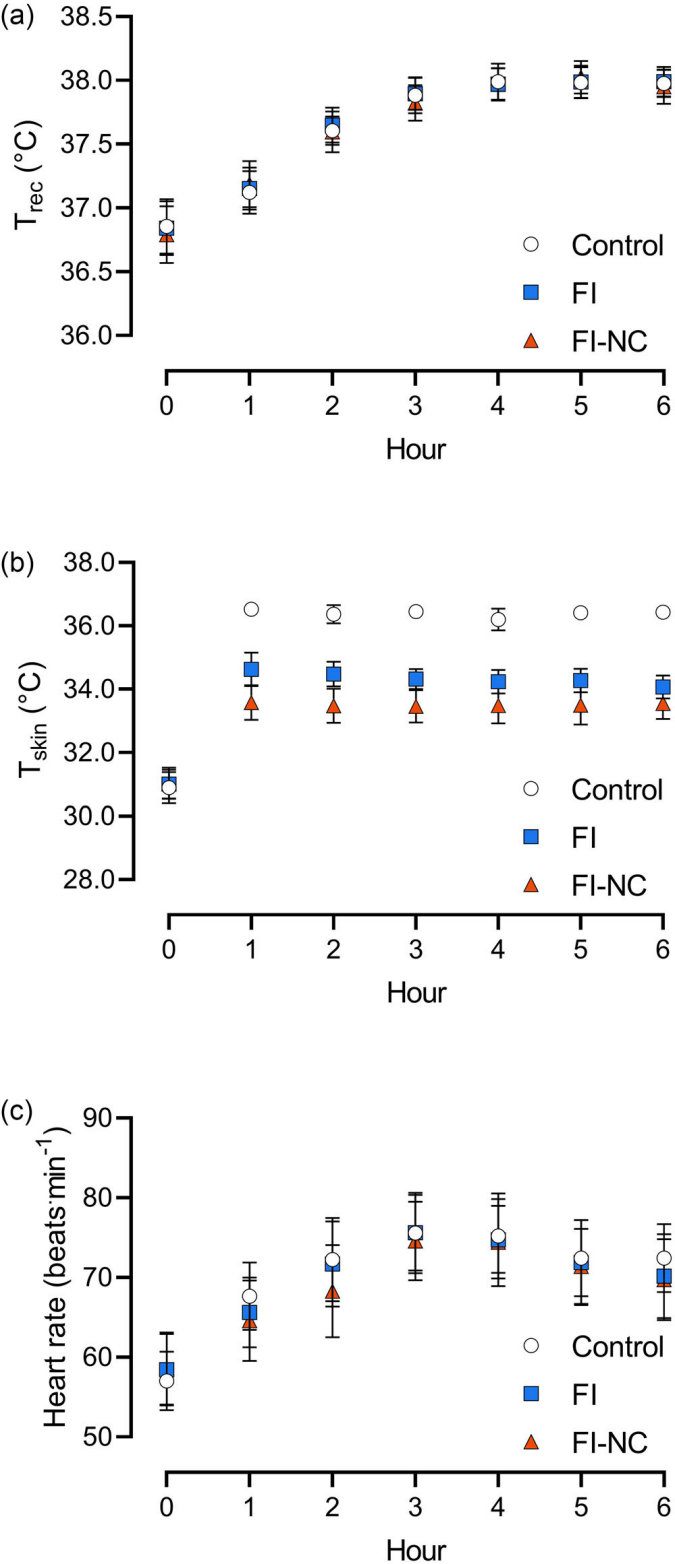
Body core temperature (a), mean skin temperature (b), and heart rate (c) during the 6 h simulated heatwave exposure with no cooling (CON; open circles), foot immersion alone (FI; blue squares), and foot immersion with neck cooling (FI‐NC; orange triangles) in 17 older adults. Data are presented as means and 95% confidence interval (error bars).

Body core temperature (Figure 2a) increased by 1.1°C (± 0.4) from baseline (hour 0) to end‐exposure (hour 6) across conditions (*P *< 0.001). Baseline‐adjusted end‐exposure body core temperature was not different between control and foot immersion (mean difference: −0.0°C [−0.1, 0.1]; *P *> 0.999) or control and foot immersion with neck cooling (mean difference: 0.0°C [−0.1, 0.1]; *P *> 0.999). Mean skin temperature increased from baseline to end‐exposure in all conditions (mean increase: 3.7 ± 0.8°C; *P *< 0.001). Baseline‐adjusted end‐exposure mean skin temperature was 2.4°C [1.9, 2.9; *P *< 0.001] greater in control compared to foot immersion, and 2.9°C [2.4, 3.4; *P *< 0.001] greater in control relative to foot immersion with neck cooling (Figure 2b). Heart rate increased from baseline to end‐exposure in all conditions (mean increase: 13 ± 4 beats/min; *P *< 0.001). Baseline‐adjusted end‐exposure heart rate was 4 beats/min [1, 7; *P* = 0.016] greater in control compared to foot immersion, and 4 beats/min [1, 7; *P *= 0.005] greater in control relative to foot immersion with neck cooling (Figure 2c).

Whole‐body sweat rate was 63 mL/h [32, 94] greater in control compared to foot immersion (*P *< 0.001) and 108 mL/h [77, 139] greater in control compared to foot immersion with neck cooling (*P *< 0.001). Fluid consumption was 59 mL/h [19, 99] greater in control compared to foot immersion (*P *< 0.001), and 77 mL/h [37, 117] greater in control compared to foot immersion with neck cooling (*P *< 0.001). Fluid loss (percentage of body mass change) was not different between control (−0.5 ± 0.9%) and foot immersion (−0.5 ± 0.9%; *P *> 0.999), but was 0.5% [0.1, 0.9] greater in control compared to foot immersion with neck cooling (*P *= 0.02). The change in plasma volume was not different between control (1.68 ± 3.73%) and foot immersion (2.43 ± 2.01%; *P *> 0.999) nor between control and foot immersion with neck cooling (1.50 ± 3.09%; *P *> 0.999).

### 3.2 Biochemical responses

**FIGURE 3:**
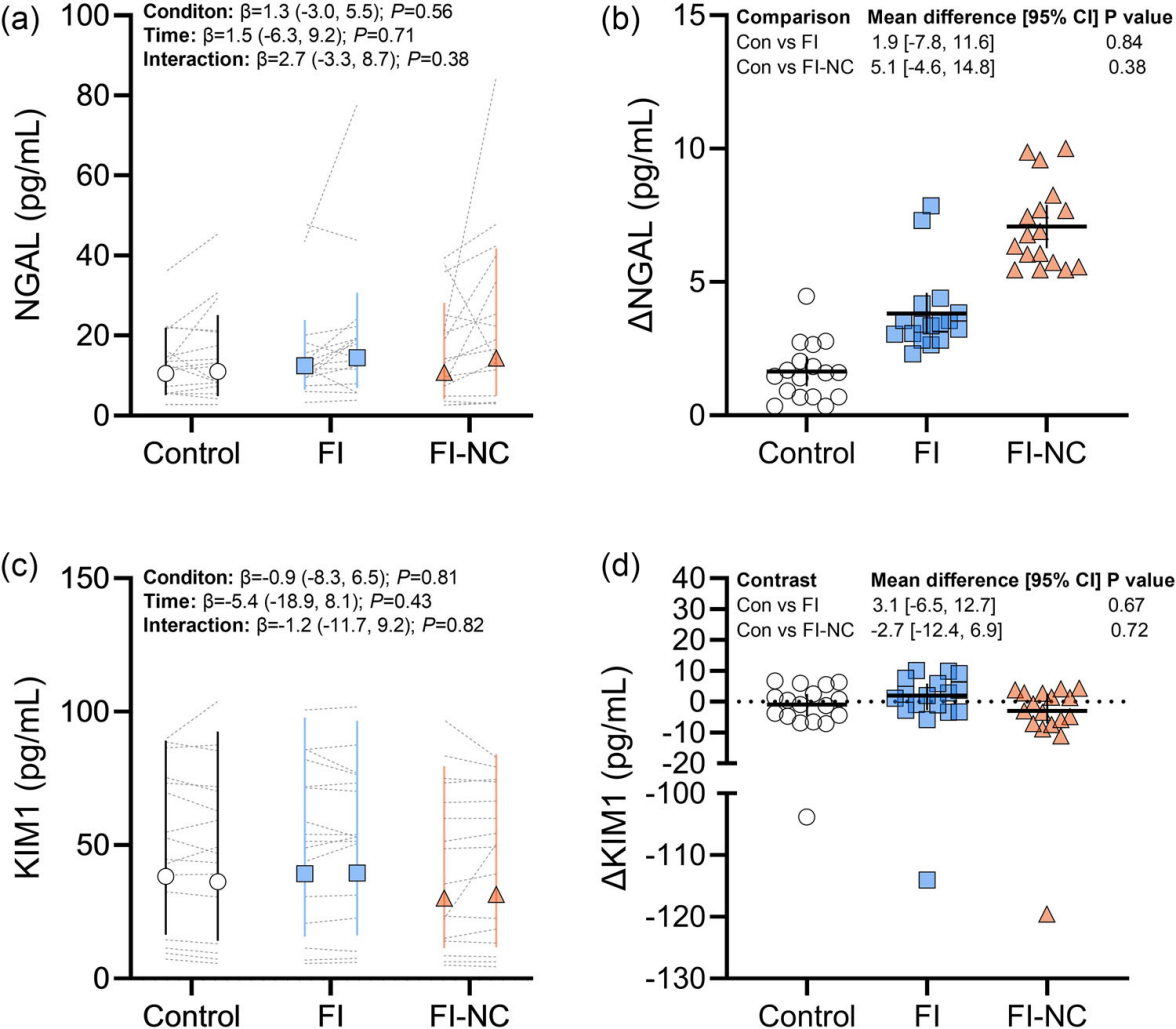
Absolute NGAL (a) and KIM1 (c) concentrations (pg/mL) at baseline and post‐exposure in the control, foot immersion (FI; blue squares), and foot immersion with neck cooling (FI‐NC; orange triangles) conditions. Individual responses are shown (dotted lines), with the geometric mean and standard deviation. Mixed linear model (MLM) outputs are reported as β‐coefficients (95% CI) and *P*‐values for fixed effects of condition, time, and their interaction. The change from baseline (Δ) in NGAL (b) and KIM1 (d) concentrations are presented as individual values (*n* = 17) with group means and 95% confidence intervals. Data show the raw‐unadjusted data and annotations present the statistical outcomes derived from the baseline adjusted linear mixed model. Pairwise contrasts (Control vs. FI, Control vs. FI‐NC) are shown with mean differences, 95% CI and *P*‐values. Individual data are shown for 17 older adults. No significant effects of condition or interaction were detected for either biomarker.

**FIGURE 4:**
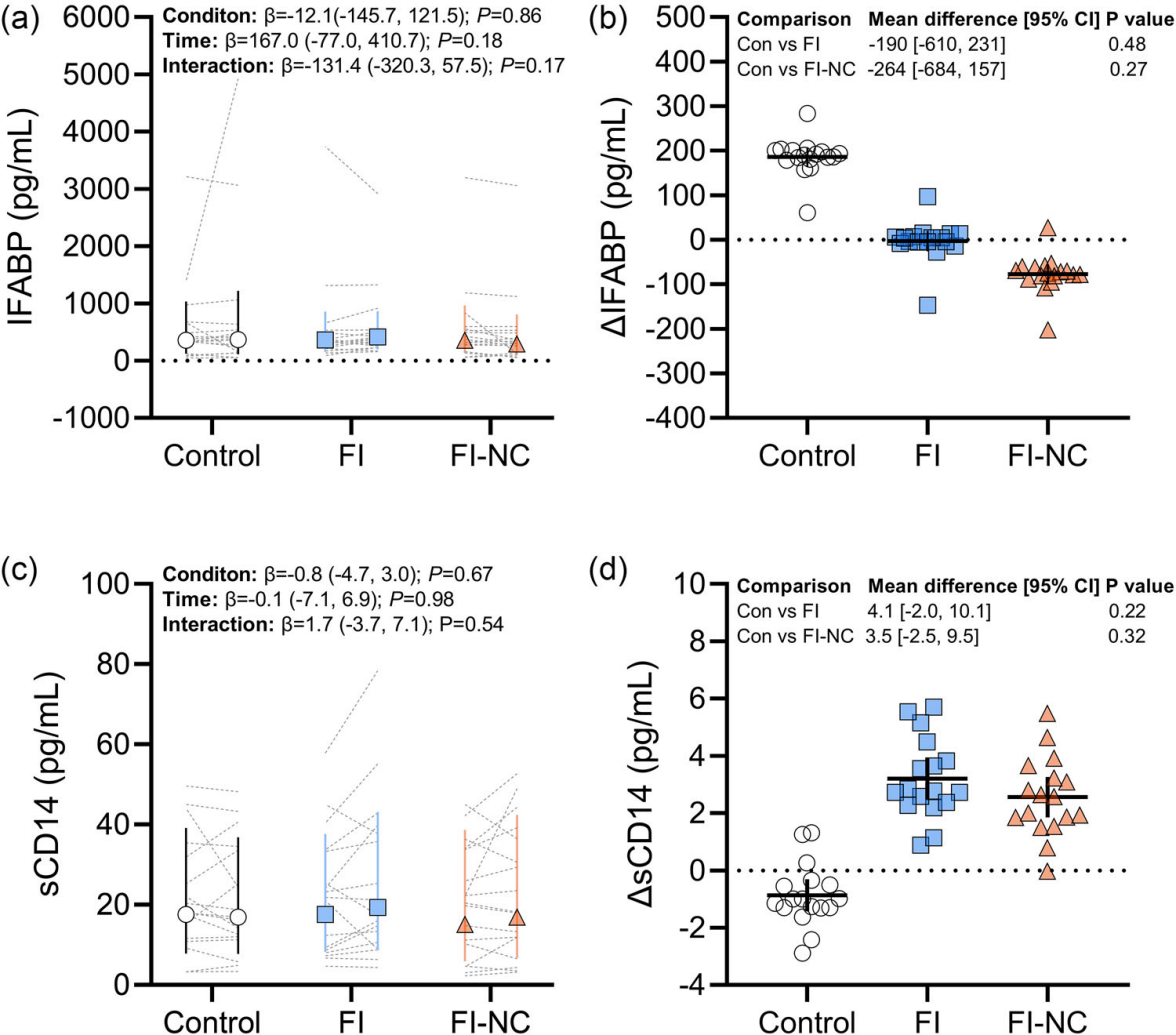
Absolute IFABP (a) and sCD14 (c) concentrations (pg/mL) at baseline and post‐exposure in the control, foot immersion (FI; blue squares), and foot immersion with neck cooling (FI‐NC; orange triangles) conditions. Individual responses are shown (dotted lines), with the geometric mean and standard deviation. Mixed linear model (MLM) outputs are reported as β‐coefficients (95% CI) and *P*‐values for fixed effects of condition, time, and their interaction. The change from baseline (Δ) in IFABP (b) and sCD14 (d) concentrations are presented as individual values (*n* = 17) with group means and 95% confidence intervals. Data show the raw‐unadjusted data and annotations present the statistical outcomes derived from the baseline adjusted linear mixed model. Pairwise contrasts (Control vs. FI, Control vs. FI‐NC) are shown with mean differences, 95% CI and *P*‐values. No significant effects of condition or interaction were detected for either biomarker.

**FIGURE 5:**
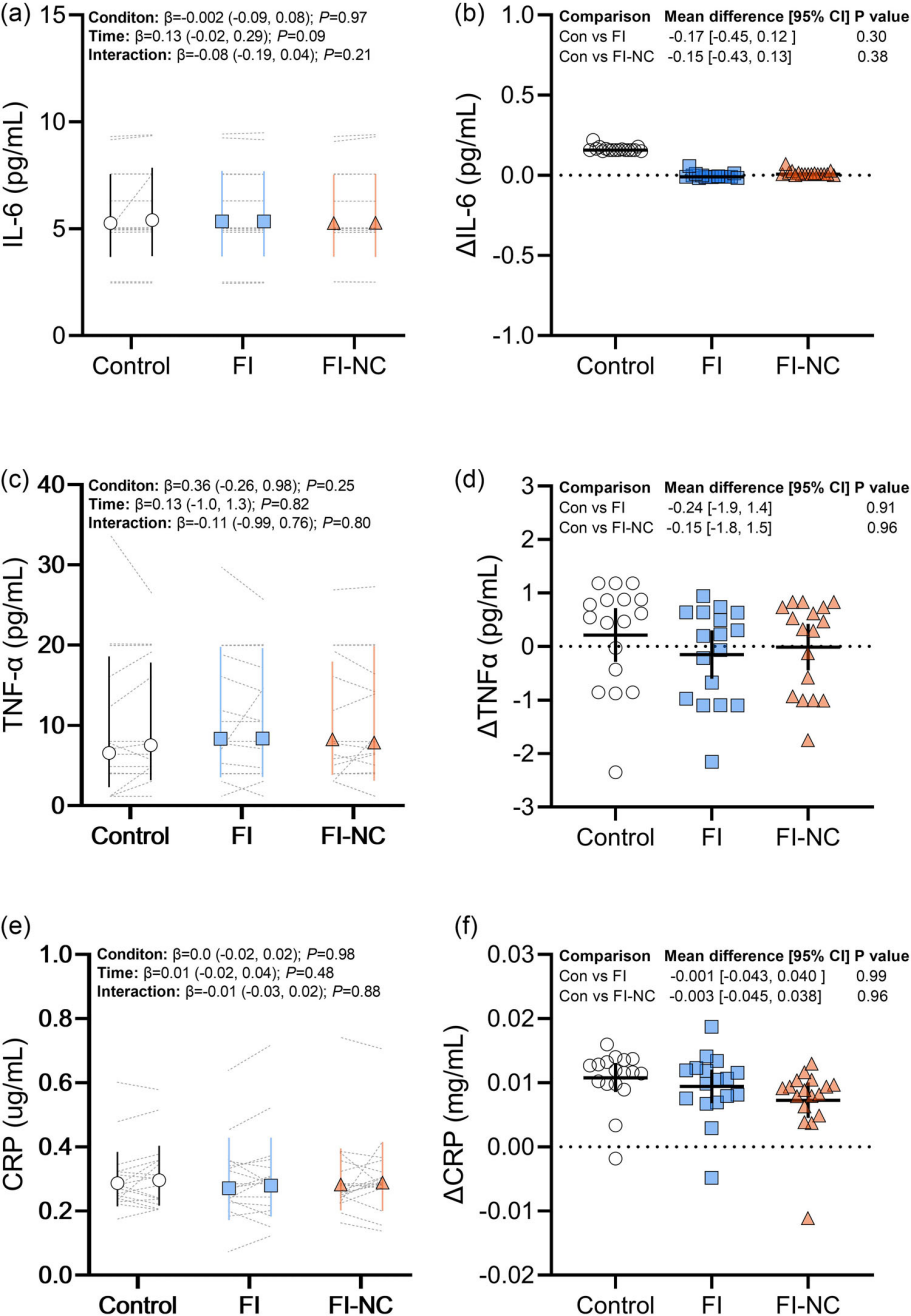
Absolute IL‐6 (a), TNF‐α (c), and CRP (e) concentrations at baseline and post‐exposure in the control, foot immersion (FI; blue squares), and foot immersion with neck cooling (FI‐NC; orange triangles) conditions. Individual responses are shown (dotted lines), with the geometric mean and standard deviation. Mixed linear model (MLM) outputs are reported as β‐coefficients (95% CI) and *P*‐values for fixed effects of condition, time, and their interaction. The change from baseline (Δ) in IL‐6 (b), TNF‐α (d), and CRP (f) concentrations are presented as individual values (*n* = 17) with group means and 95% confidence intervals. Data show the raw‐unadjusted data and annotations present the statistical outcomes derived from the baseline adjusted linear mixed model. Pairwise contrasts (Control vs. FI, Control vs. FI‐NC) are shown with mean differences, 95% CI and *P*‐values. No significant effects of condition or interaction were detected for any biomarker.

No main effects for condition and time, and no condition × time interaction were observed for NGAL, KIM1, IFABP, sCD14, IL‐6, TNF‐α or CRP (all *P *> 0.05; Figures 3, 4, 5). A main effect of time (F = 7.8, *P *= 0.013) was observed for NGAL, which increased by 4.2 pg/mL (0.8, 7.6), regardless of condition (Figure 3a). Statistical outputs are displayed within each figure panel.

The baseline adjusted post‐exposure concentrations for NGAL (Figure 3b) and KIM‐1 (Figure 3d) were not different between control and foot immersion (all *P *> 0.999), or between control and foot immersion with neck cooling (all *P *> 0.682). Similarly, the baseline adjusted post‐exposure concentrations for IFABP (Figure 4b), sCD14 (Figure 4d), IL‐6 (Figure 5b), TNF‐α (Figure 3d) and CRP (Figure 5f) were not different between control and foot immersion (all *P *> 0.22), or between control and foot immersion with neck cooling (all *P *> 0.27). The mean difference and 95% confidence intervals are displayed within each figure panel.

## 4 DISCUSSION

This study evaluated the effect of foot immersion with and without neck cooling on systemic proteins associated with acute kidney injury (NGAL, KIM‐1), intestinal enterocyte damage (IFABP), immune activation (sCD14) and systemic inflammation (IL‐6, TNF‐α, CRP) in older adults following 6 h of simulated heatwave exposure (38°C, 35% relative humidity). We found that all post‐exposure systemic protein concentrations were unchanged relative to baseline and were not affected by foot immersion with or without neck cooling, alongside a ∼1.1°C increase in body core temperature across conditions. Relative to the control, both cooling interventions reduced mean skin temperature, heart rate, whole‐body sweat rate and fluid consumption; and foot immersion with neck cooling reduced fluid loss. However, these effects were modest and unlikely clinically meaningful (Meade et al., 2024). In totality, foot immersion with or without neck cooling did not mitigate hyperthermia and had small effects on cardiovascular strain. The efficacy of these interventions for protecting against renal ischaemia or proximal tubular injury, intestinal enterocyte damage, immune activation or systemic inflammation in older adults during heatwaves requires further examination during longer, more severe and/or multi‐day exposures.

We observed no changes in plasma NGAL or KIM‐1, suggesting older adults did not experience renal ischaemia or proximal tubular injury, respectively (Schlader et al., 2019), during the 6 h exposure to conditions reflective of indoor overheating, regardless of condition. Increases in NGAL, a bacteriostatic agent involved in the immune response to ischaemia‐mediated acute kidney injury (Schlader et al., 2019), and KIM‐1, a transmembrane glycoprotein upregulated in proximal tubule cells following ischaemia–reperfusion and/or toxic kidney injury (Schlader et al., 2019), appear to be modulated by hyperthermia, dehydration and/or exercise/heat‐induced compensatory reductions in renal perfusion supporting increased muscle and skin blood flow demands for metabolism and heat dissipation, respectively (Chapman, Johnson, Vargas, et al., 2020; Chapman et al., 2023; Houck et al., 2022; Juett et al., 2024; Lee, Flood, Russell, et al., 2024; Schlader et al., 2017). The rise in body core temperature (∼1.1°C), limited dehydration (≤0.5% body mass loss) and lack of physical activity in the present study were likely insufficient to elicit measurable renal ischaemia or proximal tubular injury. Moderate exercise in the heat, resulting in greater magnitudes of hyperthermia (∼1.9°C increase in body core temperature) and dehydration (∼1.46% body mass loss) than in the present study, was shown to elevate both plasma NGAL and KIM‐1 in older adults (Lee, Flood, Russell, et al., 2024). Thus, longer and/or more severe (e.g., temperature, humidity) passive heat exposures may be required to elicit measurable renal ischaemia and/or proximal tubular damage, for which the effect of foot immersion and neck cooling could be evaluated in future research.

In line with this, a recent study by Lee et al. (2025b) reported small ∼3.7 pg/mL increases in plasma NGAL following 9 h of passive heat exposure (40.3°C, 9.3% relative humidity). In that study, moving to an air‐conditioned room (23°C, 50% relative humidity) for 2 h during the second half of the exposure reduced plasma NGAL by ∼3.2 pg/mL relative to no‐cooling (Lee et al., 2025b). While this suggests that air‐conditioned environments (e.g., cooling centres) may mitigate renal ischaemia and in turn reduce the risk for acute kidney injury in older adults during heatwaves, it is important to note that the expression of plasma NGAL is not specific to the kidneys and thus elevations in this biomarker are not directly reflective of increased acute kidney injury risk (Chakraborty et al., 2012; Chapman, Johnson, Parker, et al., 2020). Moreover, air‐conditioned environments may be inaccessible for some older adults (e.g., due to limited mobility, lack of transportation, financial constraints, etc.; Bedi et al., 2022; Kenny et al., 2024). While it remains unknown whether foot immersion alone or with neck cooling would have mitigated elevations in NGAL or KIM‐1 under more severe exposure conditions, this appears unlikely since the cooling interventions did not lower body core temperature and only modestly reduced cardiovascular strain, especially considering that greater sweat rates and fluid losses in the control were almost entirely offset by greater fluid consumption (Meade et al., 2024), underscoring the importance of maintaining hydration during heat stress. Nevertheless, this highlights the need for future experiments involving longer, more severe and/or repeated heat exposures, to develop effective practical and sustainable alternatives to electrical cooling systems.

We observed no changes in the biomarkers associated with intestinal enterocyte damage (IFABP), immune activation (sCD14), or systemic inflammation (TNF‐α, IL‐6, CRP) – pathophysiological events linked to the development of heatstroke (Leon & Helwig, 2010; Ogden et al., 2020; Schlader et al., 2022; Ye et al., 2019; Zhang et al., 2024) – following the 6 h passive exposure to extreme heat. The magnitude of hyperthermia and/or cardiovascular strain elicited during the exposure were likely insufficient to initiate these pathophysiological events, since both hyperthermia and cardiovascular strain can augment intestinal hyperpermeability (Pires et al., 2017; Walter et al., 2021), and elevations in body core temperature is predictive of changes to IFABP, sCD14 and downstream systemic inflammatory markers (e.g., TNFα, IL‐1β, IL‐6, IL‐8, IL‐10 and IL‐1ra) during exertional heat stress (Henningsen et al., 2023). Consequently, it remains unknown whether foot immersion and neck cooling would protect against these pathophysiological events under more severe conditions, though it appears unlikely given the limited effects these interventions had on thermal and cardiovascular strain.

In contrast with our present findings, recent studies have shown older adults to experience elevated IFABP following both active (Foster et al., 2023; Lee, Flood, Galan‐Lopez, et al., 2024) and passive (Lee, Russell, et al., 2024; Lee et al., 2025b, 2025a) simulated heatwave exposures. However, these elevations in IFABP seldom exceeded the proposed threshold for clinical significance (change in IFABP >1000 pg/mL; Costa et al., 2017), which may explain why intestinal enterocyte damage rarely translated into elevated markers of immune activation or systemic inflammation (Foster et al., 2023; Lee, Flood, Galan‐Lopez, et al., 2024; Lee, Russell, et al., 2024; Lee et al., 2025a). The absence of IFABP elevation in the present study could relate to the shorter duration (6 h) and arguably less severe environmental conditions (38°C, 35% relative humidity) of the passive exposure relative to those employed by Lee, Russell, et al. (2024) and Lee et al. (2025b): 9 h at ∼40°C, ∼9% relative humidity. Under those conditions, older adults experienced a progressive rise in body core temperature, with hyperthermia plateauing after ∼6 h of exposure (Meade et al., 2023), and small but significant elevations in post‐exposure IFABP (Lee, Russell, et al., 2024). Although the magnitude of hyperthermia was numerically greater in the present study (∼1.1°C) than in Lee, Russell, et al. (2024), ∼0.9°C, and Lee et al. (2025b), ∼0.8°C, the longer duration of sustained hyperthermia (i.e., from 6–9 h) in those studies may have contributed to the significant, albeit clinically trivial, elevations in IFABP. Furthermore, the lack of relationship between changes in body core temperature and changes in IFABP during 8 h of passive heat exposure (36°C, 45% relative humidity) in older adults (Lee et al., 2025a) may suggest that the duration of sustained hyperthermia plays a more influential role than its magnitude in eliciting detectable intestinal enterocyte damage. This highlights the need to examine longer duration heat exposures both acutely (i.e., a full day) and more chronically (repeated day and overnight exposures), also incorporating activities reflective of daily living, to determine whether foot immersion and neck cooling mitigate enterocyte damage and reduces heatstroke risk under more severe conditions. It is noteworthy that across multiple controlled simulated heatwave studies with exposure durations of 3–9 h, and conditions ranging from hot–dry (i.e., 40°C, 9% RH; Meade et al., 2023) to hot–humid (41°C, 40% RH; Mckenna et al., 2025), otherwise healthy older adults have demonstrated modest thermoregulatory strain (∼+1.0°C in core temperature) and limited evidence of renal, intestinal or systemic stress biomarkers (Lee et al., 2025b, 2025a; Meade et al., 2024, 2023; McKenna et al., 2025). This contrasts with the widely reported population‐level increases in morbidity and mortality among older adults during real‐world heat events. One plausible explanation is that vulnerability may be concentrated among clinically compromised individuals (e.g., with multimorbidity, reduced functional capacity or social/environmental disadvantages), rather than among all older adults per se. Future research should therefore aim to directly compare physiological and biomarker responses between healthy and clinically vulnerable subgroups, to better identify who is most at risk and to inform targeted intervention strategies.

### 4.1 Limitations

The present study has several limitations that warrant further consideration. Firstly, the generalizability of our findings are limited to one specific set of environmental conditions (38°C, 35% relative humidity) which, despite simulating an overheated dwelling during recent deadly heatwaves in North America (Henderson et al., 2022), may not reflect the broader range of conditions conducive to heat stress (e.g., very hot, dry; warm, very humid) experienced by older adults globally. Thus, the potential efficacy of foot immersion with or without neck cooling under alternate (e.g., hot, dry vs. warm, wet), or more severe temperature/humidity combinations remains unknown. Employing longer and/or repeated exposures would more accurately reflect the physiological consequences experienced by older adults during heatwaves, defined as prolonged periods of excessive heat (Perkins‐Kirkpatrick & Lewis, 2020). Secondly, we were unable to directly measure gastrointestinal permeability (e.g., via the dual‐sugar absorption test with lactulose and l‐rhamnose; Ogden et al., 2020). Recent research has demonstrated older adults experience increases in intestinal permeability but not IFABP or sCD14 during controlled passive hyperthermia (McKenna et al., 2025). Studies which directly measure intestinal permeability are therefore required to ascertain the effects of foot immersion with or without neck cooling on gastrointestinal function and heatstroke risk in older adults. Thirdly, our interpretation of acute kidney injury risk was limited by our analysis of plasma NGAL and KIM‐1, which lacks specificity for detecting acute kidney injury risk due to their expression in organs other than the kidneys (Juett et al., 2020). Measuring the urinary forms of these biomarkers provides additional insight into the location of renal damage (e.g., proximal and/or distal tubules, glomerulus), severity of renal damage, as well as the mechanisms underlying heat stress‐induced renal damage and increased acute kidney injury risk in older adults (Chapman, Johnson, Parker, et al., 2020). Such information would aid in enhancing the development of practical and sustainable cooling interventions for protecting older adults’ renal health and function during heatwaves, whilst also providing additional mechanistic insights regarding renal function in older adults exposed to extreme heat. Finally, venous blood was only collected before and after the 6 h heat exposure, precluding the assessment of the time course of biomarker responses, and may have missed transient changes occurring earlier in the exposure that were not evident at the group level post‐exposure. It is also feasible that the profile of biomarker changes may also peak later in the post‐exposure period at a time point not captured by one‐off spot sampling. Future studies incorporating serial blood sampling are warranted to determine whether non‐electrical cooling interventions alter the temporal pattern of pathophysiological responses during heat stress. Research involving longer and/or repeated simulated heatwave exposures across a range of conditions (e.g., very hot, dry vs. hot, humid), and incorporating a more comprehensive range of renal and gastrointestinal function/tissue injury measurement techniques, are also warranted.

### 4.2 Conclusion

There were no changes in the measured systemic proteins associated with renal ischaemia or proximal tubular injury, intestinal enterocyte damage, immune activation or systemic inflammation, regardless of condition. These findings do not support the efficacy of foot immersion with or without neck cooling for protecting older adults without access to electrical cooling systems from heat‐related pathophysiological outcomes during heatwaves.

## AUTHOR CONTRIBUTIONS

Glen P. Kenny, Robert D. Meade, James J. McCormick, and Ben J. Lee conceptualized and designed the research; Kelli E. King, James J. McCormick, and Emma R. McCourt performed data collection; James J. McCormick performed the blood analysis; Thomas McCarthy, and Ben J. Lee, performed statistical analysis and prepared figures; Thomas McCarthy and Ben J. Lee drafted the manuscript; all authors interpreted the results; all authors edited and revised the final version. All authors have read and approved the final version of this manuscript and agree to be accountable for all aspects of the work in ensuring that questions related to the accuracy or integrity of any part of the work are appropriately investigated and resolved. All persons designated as authors qualify for authorship, and all those who qualify for authorship are listed.

## CONFLICT OF INTEREST

No conflict of interest, financial or otherwise, are declared by the authors.

## Data Availability

Data is available by the corresponding author upon reasonable request.

## References

[eph70104-bib-0002] Bedi, N. S. , Adams, Q. H. , Hess, J. J. , & Wellenius, G. A. (2022). The role of cooling centers in protecting vulnerable individuals from extreme heat. Epidemiology, 33(5), 611–615. 35706096 10.1097/EDE.0000000000001503 PMC9378433

[eph70104-bib-0004] Chakraborty, S. , Kaur, S. , Guha, S. , & Batra, S. K. (2012). The multifaceted roles of neutrophil gelatinase associated lipocalin (NGAL) in inflammation and cancer. Biochimica et Biophysica Acta, 1826(1), 129–169. 22513004 10.1016/j.bbcan.2012.03.008 PMC3362670

[eph70104-bib-0005] Chapman, C. L. , Holt, S. M. , O'Connell, C. T. , Brazelton, S. C. , Howells, W. A. B. , Medved, H. N. , Reed, E. L. , Needham, K. W. , Halliwill, J. R. , & Minson, C. T. (2023). Acute kidney injury biomarkers and hydration assessments following prolonged mild hypohydration in healthy young adults. American Journal of Physiology. Renal Physiology, 325(2), F199–F213. 37318992 10.1152/ajprenal.00086.2023 PMC10396285

[eph70104-bib-0006] Chapman, C. L. , Johnson, B. D. , Parker, M. D. , Hostler, D. , Pryor, R. R. , & Schlader, Z. (2020). Kidney physiology and pathophysiology during heat stress and the modification by exercise, dehydration, heat acclimation and aging. Temperature, 8(2), 108–159. 10.1080/23328940.2020.1826841 PMC8098077 33997113

[eph70104-bib-0007] Chapman, C. L. , Johnson, B. D. , Vargas, N. T. , Hostler, D. , Parker, M. D. , & Schlader, Z. J. (2020). Both hyperthermia and dehydration during physical work in the heat contribute to the risk of acute kidney injury. Journal of Applied Physiology, 128(4), 715–728. 32078468 10.1152/japplphysiol.00787.2019 PMC7191500

[eph70104-bib-0008] Costa, R. J. S. , Snipe, R. M. J. , Kitic, C. M. , & Gibson, P. R. (2017). Systematic review: Exercise‐induced gastrointestinal syndrome‐implications for health and intestinal disease. Alimentary Pharmacology & Therapeutics, 46(3), 246–265. 28589631 10.1111/apt.14157

[eph70104-bib-0055] Dill, D. B. , & Costill, D. L. (1974). Calculation of percentage changes in volumes of blood, plasma, and red cells in dehydration. Journal of Applied Physiology, 37(2), 247–248. 4850854 10.1152/jappl.1974.37.2.247

[eph70104-bib-0012] Foster, J. , Mckenna, Z. J. , Atkins, W. C. , Jarrard, C. P. , & Crandall, C. G. (2023). Aging increases enterocyte damage during a 3‐hour exposure to very hot and dry heat: A preliminary study. Biology, 12(8), 1088. 37626974 10.3390/biology12081088 PMC10451985

[eph70104-bib-0013] Gasparrini, A. , Guo, Y. , Hashizume, M. , Lavigne, E. , Tobias, A. , Zanobetti, A. , Schwartz, J. D. , Leone, M. , Michelozzi, P. , Kan, H. , Tong, S. , Honda, Y. , Kim, H. , & Armstrong, B. G. (2016). Changes in susceptibility to heat during the summer: A multicountry analysis. American Journal of Epidemiology, 183(11), 1027–36. 27188948 10.1093/aje/kwv260 PMC4887574

[eph70104-bib-0014] Henderson, S. B. , McLean, K. E. , Lee, M. J. , & Kosatsky, T. (2022). Analysis of community deaths during the catastrophic 2021 heat dome: Early evidence to inform the public health response during subsequent events in greater Vancouver, Canada. Environmental Epidemiology, 6(1), e189. 35169667 10.1097/EE9.0000000000000189 PMC8835552

[eph70104-bib-0015] Henningsen, K. , Mika, A. , Alcock, R. , Gaskell, S. K. , Parr, A. , Rauch, C. , Russo, I. , Snipe, R. M. J. , & Costa, R. J. S. (2023). The increase in core body temperature in response to exertional‐heat stress can predict exercise‐induced gastrointestinal syndrome. Temperature, 11(1), 72–91. 10.1080/23328940.2023.2213625 PMC10989703 38577295

[eph70104-bib-0016] Houck, J. , McKenna, Z. , Fennel, Z. , Ducharme, J. , Wells, A. , Mermier, C. , Deyhle, M. , Laitano, O. , Specht, J. , & Amorim, F. (2022). The effect of interval and continuous work on markers of acute kidney injury in a hot environment. European Journal of Applied Physiology, 122(11), 2437–2450. 35999474 10.1007/s00421-022-05030-1

[eph70104-bib-0017] Jay, O. , Capon, A. , Berry, P. , Broderick, C. , de Dear, R. , Havenith, G. , Honda, Y. , Kovats, R. S. , Ma, W. , Malik, A. , Morris, N. B. , Nybo, L. , Seneviratne, S. I. , Vanos, J. , & Ebi, K. L. (2021). Reducing the health effects of hot weather and heat extremes: From personal cooling strategies to green cities. The Lancet, 398(10301), 709–724. 10.1016/S0140-6736(21)01209-5 34419206

[eph70104-bib-0019] Juett, L. A. , Drury, J. E. , Greensmith, T. B. , Thompson, A. P. , Funnell, M. P. , James, L. J. , & Mears, S. A. (2024). Hypohydration induced by prolonged cycling in the heat increases biomarkers of renal injury in males. European Journal of Applied Physiology, 124(4), 1085–1096. 37848571 10.1007/s00421-023-05328-8 PMC10954877

[eph70104-bib-0020] Juett, L. A. , James, L. J. , & Mears, S. A. (2020). Effects of exercise on acute kidney injury biomarkers and the potential influence of fluid intake. Annals of Nutrition & Metabolism, 76(Suppl 1), 53–59. 33774615 10.1159/000515022

[eph70104-bib-0021] Kenefick, R. W. , & Cheuvront, S. N. (2012). Hydration for recreational sport and physical activity. Nutrition Reviews, 70(Suppl 2), S137–S142. 23121349 10.1111/j.1753-4887.2012.00523.x

[eph70104-bib-0022] Kenny, G. P. , Tetzlaff, E. J. , Journeay, W. S. , Henderson, S. B. , & O'Connor, F. K. (2024). Indoor overheating: A review of vulnerabilities, causes, and strategies to prevent adverse human health outcomes during extreme heat events. Temperature, 11(3), 203–246. 10.1080/23328940.2024.2361223 PMC11346563 39193048

[eph70104-bib-0025] Kohl, H. W. , Blair, S. N. , Paffenbarger, R. S., Jr , Macera, C. A. , & Kronenfeld, J. J. (1988). A mail survey of physical activity habits as related to measured physical fitness. American Journal of Epidemiology, 127(6), 1228–1239. 3369421 10.1093/oxfordjournals.aje.a114915

[eph70104-bib-0026] Lee, B. , Meade, R. , Davey, S. , Thake, C. , McCormick, J. , King, K. , & Kenny, G. P. (2025b). Effect of brief ambient cooling on serum stress biomarkers in older adults during a daylong heat exposure: A laboratory‐based heat wave simulation. Applied Physiology, Nutrition, and Metabolism, 50, 1–8. 10.1139/apnm-2024-0476 40036754

[eph70104-bib-0027] Lee, B. , Meade, R. D. , Davey, S. L. , Thake, C. D. , McCormick, J. J. , King, K. E. , & Kenny, G. P. (2025a). Effects of daylong exposure to indoor overheating on enterocyte damage and inflammatory responses in older adults: A randomized crossover trial. Applied Physiology, Nutrition, and Metabolism, 50, 1–7. 10.1139/apnm-2024-0368 39869857

[eph70104-bib-0028] Lee, B. J. , Flood, T. R. , Galan‐Lopez, N. , McCormick, J. J. , King, K. E. , Fujii, N. , & Kenny, G. P. (2024). Changes in surrogate markers of intestinal epithelial injury and microbial translocation in young and older men during prolonged occupational heat stress in temperate and hot conditions. European Journal of Applied Physiology, 124(4), 1049–1062. 37815618 10.1007/s00421-023-05329-7

[eph70104-bib-0029] Lee, B. J. , Flood, T. R. , Russell, S. L. , McCormick, J. J. , Fujii, N. , & Kenny, G. P. (2024). Impacts of age, type 2 diabetes, and hypertension on circulating neutrophil gelatinase‐associated lipocalin and kidney injury molecule‐1 after prolonged work in the heat in men. European Journal of Applied Physiology, 124(10), 2923–2939. 38753017 10.1007/s00421-024-05505-3

[eph70104-bib-0030] Lee, B. J. , Russell, S. L. , Meade, R. D. , McCormick, J. J. , King, K. E. , & Kenny, G. P. (2024). Markers of enterocyte damage, microbial translocation, and systemic inflammation following 9 h of heat exposure in young and older adults. Applied Physiology, Nutrition, and Metabolism, 49(9), 1241–1251. 10.1139/apnm-2024-0094 38772045

[eph70104-bib-0031] Leon, L. R. , & Helwig, B. G. (2010). Heat stroke: Role of the systemic inflammatory response. Journal of Applied Physiology (1985), 109(6), 1980–1988. 10.1152/japplphysiol.00301.2010 20522730

[eph70104-bib-0033] Lim, C. L. (2018). Heat sepsis precedes heat toxicity in the pathophysiology of heat stroke‐a new paradigm on an ancient disease. Antioxidants, 7(11), 149. 30366410 10.3390/antiox7110149 PMC6262330

[eph70104-bib-0034] McCourt, E. R. , Meade, R. D. , Richards, B. J. , Koetje, N. J. , Santucci, N. B. , McCormick, J. J. , Boulay, P. , Sigal, R. J. , & Kenny, G. P. (2024). The effect of foot immersion and neck cooling on cardiac autonomic function in older adults exposed to indoor overheating: A randomized crossover trial. Applied Physiology, Nutrition, and Metabolism, 49(12), 1773–1782. 10.1139/apnm-2024-0126 39137443

[eph70104-bib-0035] McKenna, Z. J. , Atkins, W. C. , Wallace, T. , Jarrard, C. P. , Crandall, C. G. , & Foster, J. (2025). Gastrointestinal permeability and kidney injury risk during hyperthermia in young and older adults. Experimental Physiology, 110(1), 79–92. 39417775 10.1113/EP092204 PMC11689130

[eph70104-bib-0036] Meade, R. D. , Akerman, A. P. , Notley, S. R. , McGinn, R. , Poirier, P. , Gosselin, P. , & Kenny, G. P. (2020). Physiological factors characterizing heat‐vulnerable older adults: A narrative review. Environment International, 144, 105909. 32919284 10.1016/j.envint.2020.105909

[eph70104-bib-0037] Meade, R. D. , McCourt, E. R. , McCormick, J. J. , Boulay, P. , Sigal, R. J. , & Kenny, G. P. (2024). Body core temperature after foot immersion and neck cooling in older adults exposed to extreme heat. The Journal of the American Medical Association, 331(3), 253–256. 38127341 10.1001/jama.2023.24417 PMC10739084

[eph70104-bib-0038] Meade, R. D. , Notley, S. R. , Akerman, A. P. , McGarr, G. W. , Richards, B. J. , McCourt, E. R. , King, K. E. , McCormick, J. J. , Boulay, P. , Sigal, R. J. , & Kenny, G. P. (2023). Physiological responses to 9 hours of heat exposure in young and older adults. Part I: Body temperature and hemodynamic regulation. Journal of Applied Physiology, 135(3), 673–687. 37439239 10.1152/japplphysiol.00227.2023

[eph70104-bib-0039] Morris, N. B. , Gruss, F. , Lempert, S. , English, T. , Hospers, L. , Capon, A. , & Jay, O. (2019). A Preliminary Study of the Effect of Dousing and Foot Immersion on Cardiovascular and Thermal Responses to Extreme Heat. The Journal of the American Medical Association, 322(14), 1411–1413. 31593262 10.1001/jama.2019.13051 PMC7015226

[eph70104-bib-0040] Muntner, P. , Shimbo, D. , Carey, R. M. , Charleston, J. B. , Gaillard, T. , Misra, S. , Myers, M. G. , Ogedegbe, G. , Schwartz, J. E. , Townsend, R. R. , Urbina, E. M. , Viera, A. J. , White, W. B. , & Wright, J. T., Jr (2019). Measurement of blood pressure in humans: a scientific statement from the American Heart Association. Hypertension, 73(5), e35–e66. 30827125 10.1161/HYP.0000000000000087 PMC11409525

[eph70104-bib-0041] Ogden, H. B. , Child, R. B. , Fallowfield, J. L. , Delves, S. K. , Westwood, C. S. , & Layden, J. D. (2020). The Gastrointestinal exertional heat stroke paradigm: pathophysiology, assessment, severity, aetiology and nutritional countermeasures. Nutrients, 12(2), 537. 32093001 10.3390/nu12020537 PMC7071449

[eph70104-bib-0043] Perkins‐Kirkpatrick, S. E. , & Lewis, S. C. (2020). Increasing trends in regional heatwaves. Nature Communications, 11(1), 3357. 10.1038/s41467-020-16970-7 PMC7334217 32620857

[eph70104-bib-0044] Pires, W. , Veneroso, C. E. , Wanner, S. P. , Pacheco, D. A. S. , Vaz, G. C. , Amorim, F. T. , Tonoli, C. , Soares, D. D. , & Coimbra, C. C. (2017). Association between exercise‐induced hyperthermia and intestinal permeability: A systematic review. Sports medicine, 47(7), 1389–1403. 27943148 10.1007/s40279-016-0654-2

[eph70104-bib-0047] Schlader, Z. J. , Chapman, C. L. , Sarker, S. , Russo, L. , Rideout, T. C. , Parker, M. D. , Johnson, B. D. , & Hostler, D. (2017). Firefighter work duration influences the extent of acute kidney injury. Medicine and Science in Sports and Exercise, 49(8), 1745–1753. 28272268 10.1249/MSS.0000000000001254

[eph70104-bib-0048] Schlader, Z. J. , Davis, M. S. , & Bouchama, A. (2022). Biomarkers of heatstroke‐induced organ injury and repair. Experimental Physiology, 107(10), 1159–1171. 35654394 10.1113/EP090142 PMC9529995

[eph70104-bib-0049] Schlader, Z. J. , Hostler, D. , Parker, M. D. , Pryor, R. R. , Lohr, J. W. , Johnson, B. D. , & Chapman, C. L. (2019). The potential for renal injury elicited by physical work in the heat. Nutrients, 11(9), 2087. 31487794 10.3390/nu11092087 PMC6769672

[eph70104-bib-0050] Tremblay, M. S. , Warburton, D. E. , Janssen, I. , Paterson, D. H. , Latimer, A. E. , Rhodes, R. E. , Kho, M. E. , Hicks, A. , Leblanc, A. G. , Zehr, L. , Murumets, K. , & Duggan, M. (2011). New Canadian physical activity guidelines. Applied Physiology, Nutrition, and Metabolism = Physiologie Appliquee, Nutrition Et Metabolisme, 36(1), 36–58. 21326376 10.1139/H11-009

[eph70104-bib-0051] Walter, E. , W Watt, P. , Gibson, O. R. , Wilmott, A. G. B. , Mitchell, D. , Moreton, R. , & Maxwell, N. S. (2021). Exercise hyperthermia induces greater changes in gastrointestinal permeability than equivalent passive hyperthermia. Physiological Reports, 9(16), e14945. 34409760 10.14814/phy2.14945 PMC8374382

[eph70104-bib-0052] Ye, N. , Yu, T. , Guo, H. , & Li, J. (2019). Intestinal Injury in Heat Stroke. The Journal of Emergency Medicine, 57(6), 791–797. 31708310 10.1016/j.jemermed.2019.08.033

[eph70104-bib-0053] Zhang, Z. , Wu, X. , Zou, Z. , Shen, M. , Liu, Q. , Zhangsun, Z. , Zhao, H. , Lei, W. , Wang, Z. , Dong, Y. , & Yang, Y. (2024). Heat stroke: Pathogenesis, diagnosis, and current treatment. Ageing Research Reviews, 100, 102409. 38986844 10.1016/j.arr.2024.102409

